# Screening and Identification of High-Yielding Strains of Conjugated Linoleic Acid and Optimization of Conditions for the Conversion of CLA

**DOI:** 10.3390/foods13121830

**Published:** 2024-06-11

**Authors:** Cunshe Chen, Fang Tong, Ruohao Sun, Ying Zhang, Zhihua Pang, Xinqi Liu

**Affiliations:** China Food Flavor and Nutrition Health Innovation Center, Beijing Technology and Business University, Beijing 100083, China; chencs@th.btbu.edu.cn (C.C.); tongfang1522@163.com (F.T.); sunrh0310@163.com (R.S.); czhcxcxk@163.com (Y.Z.); liuxinqi@btbu.edu.cn (X.L.)

**Keywords:** conjugated linoleic acid, lactic acid bacteria, screening of bacterial species, 16SrDNA

## Abstract

Conjugated linoleic acid (CLA) is a class of naturally occurring octadecadienoic acid in humans and animals and is a general term for a group of conformational and positional isomers of linoleic acid. In order to obtain the development of excellent lactic acid strains with a high production of conjugated linoleic acid, 32 strains with a possible CLA conversion ability were obtained by initial screening using UV spectrophotometry, and then the strains were re-screened by gas chromatography, and finally, the strain with the highest CLA content was obtained. The strains were optimized for cultivation by changing the amount of substrate addition, inoculum amount, and fermentation time. The results showed that the yield of the experimentally optimized strain for the conversion of conjugated linoleic acid could reach 94.68 ± 3.57 μg/mL, which was 74.4% higher than the initial yield of 54.28 ± 2.12 μg/mL of the strain. The results of this study can provide some basis for the application of conjugated linoleic acid production by *Lactobacillus paracasei* in the fermentation of lactic acid bacteria.

## 1. Introduction

Conjugated linoleic acid (CLA) is a general term for octadecadienoic acid (ODA) containing conjugated double bonds. It is rich in isomers due to the differences in the geometrical configurations of different CLA, which depends on different positions of the double bonds on the carbon atoms [1,2]. In recent years, CLA as a functional fatty acid has been reviewed by researchers and scholars worldwide. Numerous studies have shown that it has a variety of physiological functions such as preventing atherosclerosis, regulating body immunity, reducing fat deposition, and promoting bone formation [3,4,5,6]. Natural CLA is mainly derived from ruminant meat products, dairy products, vegetable oil products, and some seafood [7,8]. However, the CLA content of such foods is very low and does not meet the recommended healthy amount of CLA for in vitro intake by humans [9], so it has become a research priority to explore how to obtain CLA yield by synthesizing it in vitro. Currently, common synthesis methods include biosynthesis, chemical synthesis, and microbial transformation, but due to the complex composition of isomers in the products, isolation and purification is relatively difficult, the risk factor is large and the products contain toxic substances; thus, their application in food and other fields has received limitations [10,11]. In contrast, the biosynthesis method of conjugated linoleic acid production through microbial conversion has mild reaction conditions, and single isomers are produced, which are easy to cultivate and can be directly used in food, nutritional and pharmaceutical products, etc. [12].

Lactic acid bacteria is an important part of animal intestinal flora [13]. In recent years, many studies have shown that lactic acid bacteria not only convert CLA but also have a high conversion rate. Deng [14] et al. investigated the ability of strains of *Lactobacillus*, *Propionibacterium*, *Bifidobacterium*, and *Enterococcus* to produce CLA, and they found that Propioni bacterium produced the highest CLA among these species. Alonso [15] et al. found that *Lactobacillus acidophilus* and *Lactobacillus casei* were able to convert free LnA into c9,t11-CLA, t10,c12-CLA and t10,t12-CLA. The lactic acid bacteria that are known to bio transform CLA are *Lactobacillus*, *Bifidobacterium*, *Lactococcus lactis*, and *Streptomyces mingitensis* [16,17,18,19]. Among them, *Lactobacillus plantarum* and *Lactobacillus acidophilus* are the most screened CLA-producing lactic acid bacteria.

In this experiment, strains with a higher CLA production capacity were isolated and screened from different varieties and brands of yogurt, kimchi, and sauerkraut, and the growth characteristics of the four strains of *Lactobacillus paracasei* screened with a higher CLA conversion capacity were investigated, and the optimization of the CLA-producing fermentation conditions was further carried out for strain 9# in order to determine the optimal fermentation conditions to produce conjugated linoleic acid. The used *Lactobacillus paracasei* can be used to produce fermented dairy products, which has certain research significance in promoting the development of conjugated linoleic acid-rich fermented products.

## 2. Materials and Methods

### 2.1. Materials

#### 2.1.1. Experimental Materials

Strain source: Different varieties and brands of yogurt and kimchi were purchased from supermarkets and online, respectively. The selected yogurt brands are as follows, respectively: Junlebao original flavor of sour milk; Amushi plain yogurt; Dongzhi yogurt; Chuanxiu yogurt; and Weidongli lactic acid bacteria original drink. The selected kimchi brands are as follows, respectively: Cuinainai spicy cabbage; Jingangshang korean pickles; Qiaotou sour kimchi; Xiaojuan pickled cabbage; and Caixaingwang Chinese sauerkraut.

Conjugated linoleic acid standard (purity ≥ 99%): NUCHEK-PREP Company (Shanghai, China). Wheat germ powder: Texas Toucan Industry and Trade Limited Company (Taian, China). Bacterial genomic DNA extraction kit: Biomiga (Shanghai, China). PCR primers 27f and 1492r were synthesized by Beijing Aoke Dingsheng Biological Science and Technology Co. (Beijing, China). The rest of the reagents were domestic and of analytical pure grade.

Activated medium: MRS medium. Identification medium: bromocresol violet medium. Fermentation medium: wheat germ medium: crude wheat germ powder 5 g, lactose 1.5 g, Tween-20 0.1 g, potassium dihydrogen phosphate 0.4 g, α-amylase 0.23 g, and 75 mL of distilled water. Stirring enzymolysis in a water bath at 60 °C took place for 1 h, and a supernatant of 50 mL was sterilized at 121 °C for 15 min.

#### 2.1.2. Instruments and Equipment

R-201 rotary evaporator (Shanghai Shengsheng Biotechnology Co., Ltd., Shanghai, China), CRG high-speed freezing centrifuge (Hitachi, Tokyo, Japan), UV2550 UV–visible spectrophotometer (Shimadzu, Tokyo, Japan), LS-B35L-III vertical pressure steam sterilizer, (Jiangyin Binjiang Medical Equipment Co., Ltd., Jiangying, China), BX51TF electron microscope (Olympus Corporation, Tokyo, Japan).

### 2.2. Test Methodh

#### 2.2.1. Sample Pretreatment

The extraction of fatty acids: we took 2 mL of the fermentation broth in a round-bottomed centrifuge tube, added 5 mL of chloroform/methanol (2:1, *v*:*v*), and vortexed and shook the mixture, letting it stand for 30 min and centrifuging it at 6000 r/min for 10 min at 4 °C, and we took out the lower layer of the organic phase with a pipette gun and put it in a screw-top centrifuge tube.

The methylation of fatty acids: Fatty acid methyl esterification was carried out by the acid–base combination method by adding 2 mL of potassium hydroxide–methanol (0.5 mol/mL) solution to the obtained organic phase, mixing well, placing the reaction in a water bath at 65 °C for 30 min, and cooling it to room temperature. Then, we added 2 mL hydrochloric acid–methanol solution (10% by volume) and placed it in a 70 °C water bath for 5 min. When it cooled to room temperature, we added 5 mL n-hexane, shaking the mixture and letting it stand, and we collected the organic layer, added anhydrous sodium sulfate to remove the water, and placed it in −20 °C to be preserved for detection.

Substrate Vegetable vegetable Oil oil Emulsionsemulsions: Linoleic acid isomerase in lactic acid bacteria is an inducible enzyme, which requires a certain amount of linoleic acid in the medium as an inducer to carry out conjugated linoleic acid synthesis [20]. Soybean oil and sunflower oil were used as fermentation substrates, and Vegetable vegetable oil and Tween 80 were added to a certain amount of distilled water at a volume ratio of 1.5:1 and dispersed by ultrasonic emulsification in an ice water bath so that the concentration of the substrate in the final substrate emulsion was 150 mg/mL, and the concentration of CLA was in the range of 82 mg/mL to 90 mg/mL. The emulsified and dispersed linoleic acid emulsion was filtered for sterilization by a sterilized microporous filter with a microporous filter membrane diameter of 0.22 μm.

#### 2.2.2. Isolation and Purification of Lactic Acid Bacteria

We took an appropriate amount of sample and added it to the MRS medium, enriching the culture medium at 37 °C for 24 h. We diluted the culture medium in a 10-fold gradient with sterile saline and took 0.2 mL of each of the three gradients of diluted bacterial solution, namely 10^−5^, 10^−6^, and 10^−7^, and added it to the surface of the MRS solid medium plate, evenly spreading it with a coating glass rod, and we made 3 parallel groups for each dilution. We placed the Petri dish upside down in a biochemical incubator and incubated it at 37 °C for 48~72 h.

After the formation of single colonies on the plate, we picked single colonies for Gram staining and conducted microscopic examinations to observe the morphology of the colonies to determine whether they were pure strains of bacteria. The specific Gram staining methods were as follows: The strain samples were coated on slides and fixed so that they were attached to the slides; the strains were stained using crystal violet staining solution, after which iodine solution was added for mordanting again, all for 1 min; decolorization was carried out using 95% ethanol until there was no purple wash-off; the staining was restained with diluted sandy yellow staining solution for about 30 s; and the slides were dried and observed under a microscope. If there were stray bacteria in the microscopic examination, then we continued to inoculate them on the MRS solid medium plate and picked a single colony again, until there were no mixed bacteria in the microscopic examination. The pure G+ bacteria were streaked and inoculated into bromocresol violet solid plate medium, and the milky white colonies with a yellow transparent circle around them were transferred to MRS slant medium and kept at 4 °C as a spare.

#### 2.2.3. Screening of CLA-Producing Lactic Acid Bacteria

The slant-preserved strains were inoculated into MRS liquid medium, incubated at 37 °C for 24 h, activated for 2 generations, and after conducting a microscopic examination to determine that there were no stray bacteria, they were obtained in wheat germ medium with an inoculum of 3% by volume fraction, incubated at 37 °C for 24 h at 120 r/min, and the fermentation broth was extracted to test the ability of the strains to convert to produce CLA. The initial screening was detected by UV spectrophotometry. After the initial screening, the strains with higher yields were selected for rescreening using gas chromatography to determine their yields.

#### 2.2.4. UV Spectrophotometric Detection of CLA

After the fermentation broth was treated according to the pretreatment method, the absorbance of each sample was measured using a 1 cm quartz cup at room temperature and 233 nm wavelength, with the uninoculated medium as a blank control. Each sample was measured three times in parallel.

#### 2.2.5. Gas Chromatography Detection Conditions

Column: capillary column DM-35ms (30 m × 0.25 mm × 0.25 μm, Dikma (Hangzhou, China)); heating program: initial temperature 150 °C, equilibrate for 3 min, 4 °C/min to 250 °C, equilibrate for 10 min. Inlet temperature 250 °C, detector temperature 250 °C, shunt ratio 20:1, injection volume 1 μL, and carrier gas is high-purity helium. Flow rate 1 mL/min. Hydrogen flame ionization detector FID, carrier gas is high-purity helium, gas is hydrogen, and combustion gas is air.

#### 2.2.6. Drawing of the CLA Standard Curve

The CLA standard was weighed and dissolved in methanol and methylated. The CLA methyl ester was diluted to different concentrations (0~230 μg/mL) with chromatographically pure hexane. The peak areas of the standard solutions at different concentrations were determined with reference to the GC detection conditions of Section 2.2.5. Taking CLA concentration as the horizontal coordinate and peak area as the vertical coordinate, the CLA standard curve was plotted.

#### 2.2.7. Morphological as Well as Physiological and Biochemical Characterization of the Strain

The screened strains were streaked and inoculated in MRS solid plate medium and cultured at 37 °C for 48 h to observe the colony characteristics, bacterial morphology, and Gram staining characteristics. The physiological and biochemical characterization of the screened strains was carried out by using physiological and biochemical test methods.

#### 2.2.8. 16S rDNA Sequence Identification of Strains

Bacterial DNA extraction: Genomic DNA was extracted using the Biomiga Bacterial Genomic DNA Extraction Kit (Shanghai, China); PCR reaction system and program (50 μL): 10 × TaqE Buffer, 5 μL; 27f (10 pmol/μL), 2.5 μL; 1492r (10 pmol/μL), 2.5 μL; dNTP (5 mmol/L), 5 μL; ddH_2_O, 27 μL. Template DNA, 7 μL. TaqE (5 U/μL), 1 μL. Primers were 27f 5′-AGAGTTTGATCCTGGCTCAG-3′ and 1492r 5′-GGTTACCTTGTTACGACTT-3′.

PCR amplification reaction conditions: pre-denaturation at 94 °C for 4 min, denaturation at 94 °C for 30 s, annealing at 58 °C for 40 s, extension at 72 °C for 90 s, 30 cycles, and finally, extension at 72 °C for 10 min.

PCR product detection: Agarose gel electrophoresis was used in this experiment to detect PCR products. The specific operation was as follows: gel preparation: 1.2% agarose solution, 1.2 g agarose heated and dissolved in 100 mL 1 × TAE Buffer, was poured into the gel plate to solidify for 30 min and electrophoresis buffer (1 × TAE Buffer) was poured into the electrophoresis tank, which was 3~5 mm higher than the gel surface. Spotting: 2–3 μL of spotting buffer (6 × loading buffer) and 5 μL of PCR product were blown evenly and then up-sampled to the agarose gel. We set the electrophoresis conditions as follows: voltage 80 V (constant voltage), current 150 mA, and time 20 min. Staining: the agarose gel after electrophoresis was immersed in 0.5 μg/mL EB solution for 12 min. UV detection: a fully automated gel imaging system was used to observe the PCR product bands.

Phylogenetic tree construction: PCR products were sequenced by Tiangen Biochemical Technology Co. (Beijing, China). Using BLAST (http://www.ncbi.nlm.nih.gov/BLAST, accessed on 28 November 2023), the 16S rDNA sequences of the determined strains were compared with the 16S of known bacterial rDNA/rRNA sequences in the GenBank database to find the species with the highest homology to the gene sequences of the screened strains with known taxonomic status. Then, the 16S rDNA gene sequences of the representative strains in the genus were extracted from GenBank/EMBL/DDBJ/LPSN databases and aligned with the sequences of the screened strains using the software MEGA4 11.0.13, and then multiple sequence comparisons were performed and the phylogenetic tree was constructed by the Neighbor-Join method. Phylogenetic tree was constructed by the “Neighbor-Join method”, and the genus of the selected strains was finally determined.

#### 2.2.9. Strain Growth and Fermentation Characterization

##### Viable Bacteria Counting Methods

In this experiment, the dilution coating method was used to calculate the number of live bacteria, the specific steps were as follows: 1 mL of bacterial solution was aspirated from the medium that reached the incubation time and added to a test tube containing 9 mL of sterile saline and mixed thoroughly to make a 10^−1^ dilution, and then, by analogy, successive bacterial dilutions of different dilutions were made of 10^−2^, 10^−3^, 10^−4^, 10^−5^, 10^−6^, and 10^−7^. We pipetted 0.1 mL of each of the dilutions of 10^−5^, 10^−6^ and 10^−7^, added them to MRS solid medium, and spread them with a sterile applicator stick. The coated plates were placed upside down in a 37 °C incubator for 2–3 days. After the colonies grew, we selected the corresponding gradient plate with 30–300 colonies to count the number of viable bacteria.

##### Strain Activation

A ring of bacterial bodies was picked from the 9# strain slant, inserted into 5 mL MRS medium, incubated at 37 °C for 24 h, inserted into MRS medium at 37 °C for 24 h for activation, according to the inoculum amount of 3% by volume fraction, and then left to be used. The viable bacteria were counted according to the dilution coating method to calculate the number of viable bacteria, and the cell concentration of the fermentation broth was adjusted to 10^8^~10^9^ cfu/mL.

##### Determination of Strain Growth Curves

The four strains, 9#, 16#, 23#, and 93#, were activated in MRS liquid medium for two generations, inoculated in MRS liquid medium at 3% inoculum, and left to incubate at 37 °C. Samples were taken at 0, 2, 4, 6, 8, 10, 12, 16, 20, 24, 28, 32, 36, and 40 h, respectively, and OD values of the fermentation broths were measured using a visible spectrophotometer at 600 nm.

##### Determination of Acidifying Capacity of Strains

The four strains, 9#, 16#, 23#, and 93#, were inoculated in MRS liquid medium at 3% inoculum and incubated at 37 °C. Samples were taken at 0, 2, 4, 6, 8, 10, 12, 16, 20, 24, 28, 32, 36, and 40 h, respectively, and the pH of the medium was measured.

##### Determination of Optimum Temperature for Strain Growth

The strains were activated in MRS liquid medium for two generations, inoculated in MRS medium with 3% inoculum, and placed in temperatures of 20 °C, 25 °C, 30 °C, 37 °C, and 40 °C for 24 h of static culture, and the OD value of the medium was measured at 600 nm.

##### Determination of Optimum pH for Strain Growth

We adjusted the pH of the MRS liquid medium to 2, 3, 4, 5, 6, 7, 8, and 9 and sterilized it at 121 °C for 15 min. The strains were activated in MRS liquid medium for two generations, inoculated into the above MRS medium with 3% inoculum, respectively, and left to incubate at 37 °C for 24 h. The OD value of the medium was measured at 600 nm.

##### Strain Salt Tolerance Assay

We added 0%, 2%, 4%, 6%, 8%, and 10% NaCl to the MRS medium and sterilized it at 121 °C and 15 min. The strains were activated in MRS liquid medium for two generations, inoculated into the above MRS medium at 3% inoculum, respectively, and incubated at 37 °C for 24 h. The OD value of the medium was measured at 600 nm.

##### Measurement of Acid Resistance

The pH 7.4 PBS buffer was adjusted to 3.0 with hydrochloric acid, the activated bacterial solution was obtained at an inoculum level of 10% after sterilization at 121 °C and 15 min, and samples were taken to determine the number of viable bacteria at 37 °C after acting for 0 min, 30 min, 60 min, 90 min, and 120 min, respectively.

#### 2.2.10. Optimization of Fermentation Conditions for CLA Production by Lactic Acid Bacteria

Based on the experiments, the four strains with better growth ability, acidification ability, acid resistance, and salt tolerance were screened for the optimization of CLA-producing fermentation conditions. After fermentation, the fermentation broth was pretreated and detected by gas chromatography. Chromatographic method and standard curve: Trace ISQ gas chromatography–mass spectrometry (GC-MS) instrument, Thermo Fisher Company, USA. Column: TR-50MS quartz capillary column (30 m × 025 mm, 0.25 µm); heating program: initial temperature 150 °C, equilibrate for 3 min, 4 °C/min to 250 °C, equilibrate for 10 min; inlet temperature 250 °C, detector temperature 250 °C, shunt ratio of 20:1, injection volume of 1 μL, high-purity helium carrier gas, flow rate of 1 mL/min.

## 3. Results

### 3.1. Results of Isolation of Lactobacilli

The colonies on the bromocresol violet plate that were selected were a creamy white or yellow color, with a diameter of 1~2 mm or slightly larger, with a larger yellow circle and typical characteristics of lactic acid bacteria, and then they were further purified by streaking on the MRS plate. The purified *Lactobacillus* strains were inoculated in MRS solid slant medium and stored in the refrigerator at 4 °C. A total of 55 strains were isolated from 29 samples, and the obtained strains were stored in slant test tubes.

### 3.2. CLA Gas Chromatography Standard Curves

**Figure 1:**
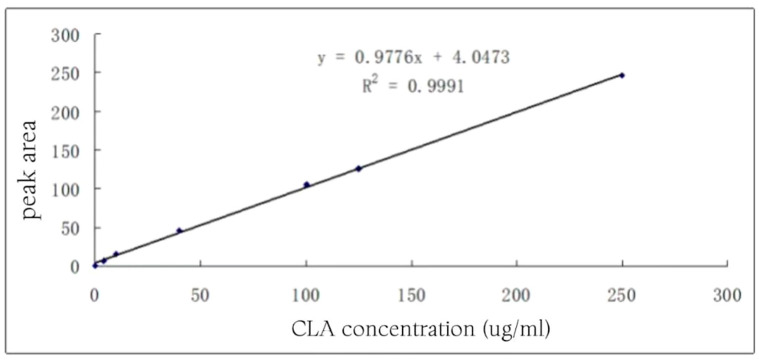
CLA standard curve.

The linear regression equation was y = 0.9776x + 4.0473, and the linear correlation coefficient was R^2^ = 0.9991. It showed that the linear relationship between the peak area and concentration was good in the concentration range of 0~250 μg/mL (Figure 1).

### 3.3. Screening of CLA-Producing Lactic Acid Bacteria

The 55 strains of *Lactobacillus casei* screened were initially screened by UV spectrophotometry, and a total of 32 strains were screened for possible CLA conversion abilities, and the absorbance values of the fermentation broth of each strain at 233 nm are shown in Table 1.

**Figure 2:**
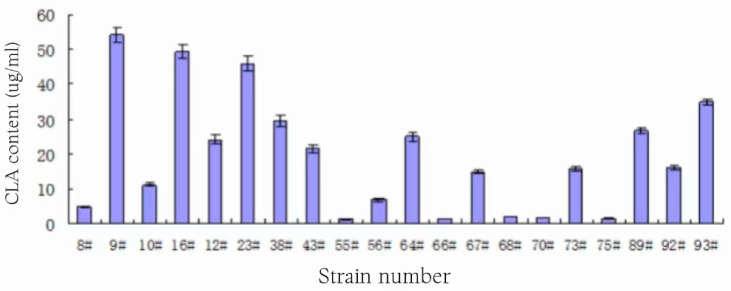
Level of CLA produced in each strain.

Gas chromatography was used to re-screen the strains with positive UV absorbance values from the initial screening, and 20 strains with CLA conversion ability were screened, and the CLA content in the fermentation broth of each strain is shown in Table 2 and Figure 2.

The CLA-producing ability of the 20 strains varied widely from Figure 2, with yields ranging from 1.28 μg/mL to 54.28 μg/mL. Nearly one-third of the strains had yields of less than 10 μg/mL, and four strains with high CLA production levels were finally screened out, namely 9#, 16#, 23#, and 93#. Among them, 16# and 23# had comparable yields of 49.29 ± 1.99 μg/mL and 45.83 ± 2.09 μg/mL, respectively; 93# had a yield of 34.82 ± 0.89 μg/mL; and 9# had the highest yield of 54.28 ± 2.12 μg/mL. Therefore, the subsequent experiments were centered on the above four strains.

### 3.4. Observations on the Morphology of Colonies and Bacteria of the Strain

The four strains 9#, 16#, 23#, and 93# with the highest yield were screened and cultured on MRS solid plate medium at 37 °C for 2 days to observe the colony morphology. Strain 9#, 23#, and 93# colonies were about 2 mm in diameter, milky white in color, rounded, with neat edges and raised centers, and had smooth, glossy, and opaque surfaces. Strain 16# colonies were slightly smaller than the other three strains, flat, slightly convex in the center, opaque, and slightly yellow in color.

**Figure 3:**
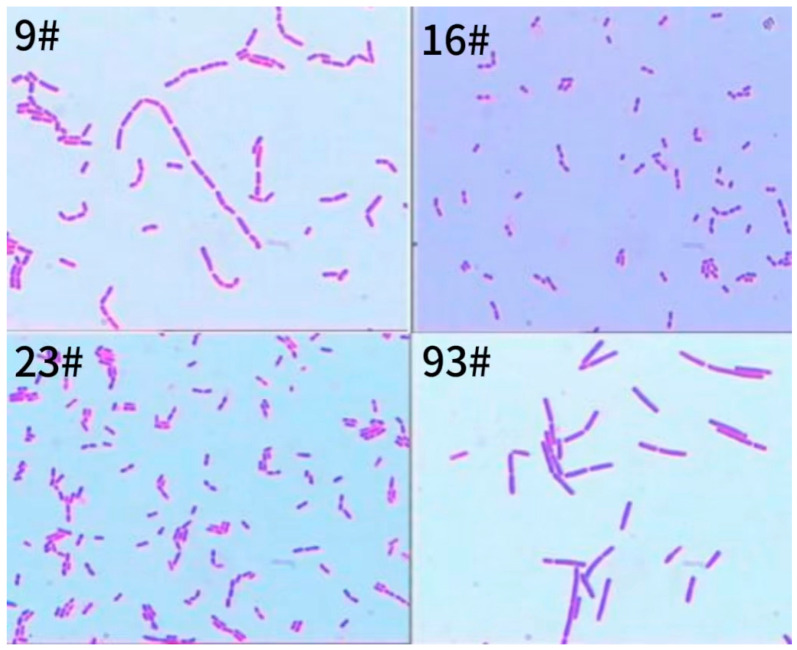
Microscopy images of the four strains.

Figure 3 shows the four strains of bacteria in the light microscope in a morphological image of the bacterium. As can be seen in the figure, strain 9# and 93# bacterial cells in a long rod did not form spores, were Gram-staining-positive, rounded at both ends, had a single or a short chain, and existed in a fenestrated arrangement. Strain 16# bacteria were short rods or globular, small, had no spores, and were Gram-positive bacteria, and most of them grew in pairs. Strain 23# had short rod-shaped cells, did not form spores, were Gram-positive, existed in single or in short chains, and were fenestrated.

### 3.5. Results of Physiological and Biochemical Characterization of Strains

The specific results of the physiological and biochemical experiments of the four strains are shown in Table 3. From the table, it can be seen that all the four strains of bacteria were positive for Gram staining, negative for catalase, and negative for the nitrate reduction test, and no budding occurred, non-liquefying gelatin was formed, and they were non-producing of hydrogen sulfide. All of these strains possessed acid-producing, decolorization, curdling, and non-liquefying properties, as shown in a litmus buttermilk test.

The results of sugar fermentation tests of the four strains are shown in Table 4. From the table, it can be seen that strains 9#, 16#, 23#, and 93# can ferment sucrose, fructose, glucose, maltose, etc., but not xylose, inulin, sorbic acid, or rhamnose, of which strain 16# could not ferment galactose, sorbitol, and mannitol compared to the other three strains.

Based on the above test results and references [21,22,23], strains 9#, 23#, and 93# were categorized as *Lactobacillus* spp., and 16# could not be identified at the time.

### 3.6. Discussion on the Analysis of the Results of Strain 16Sr DNA Sequence Identification

**Figure 4:**
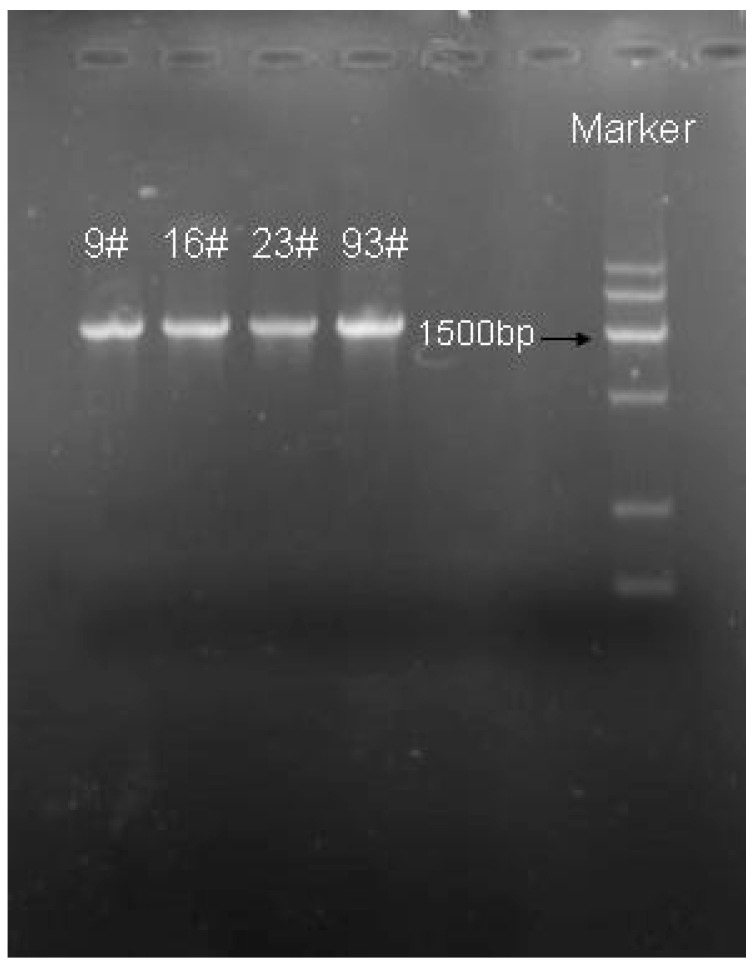
Electrophoretic map of 16s rDNA gene amplification in strain.

The DNA of the four strains was extracted separately using the Biomiga Bacterial Genomic DNA Extraction Kit before PCR amplification, and the PCR products were analyzed by agarose gel electrophoresis, and the electrophoresis results are shown in Figure 4. The PCR-amplified bands of the four strains were relatively clear, and the bands were all around 1500 bp, which were the target fragments.

#### 3.6.1. Analysis of Strain 16Sr DNA Amplification Products

The PCR products were desalted and purified and then sequenced by Beijing Tiangen Biochemical Science and Technology Co., Ltd. (Beijing, China) in both directions, and the sequences were spliced and analyzed using DNA Star software (dnastar 7.1). Moreover, the 9# sequence length was 1468 bp, the 16# sequence length was 1473 bp, the 23# sequence length was 1460 bp, and the 93# sequence length was 1471 bp.

#### 3.6.2. 16S rDNA Sequence Homology Analysis of Strains

The 16S rDNA sequences of the four strains were submitted to http://www.NCBI.nlm.nih.gov/blast, accessed on 15 December 2023, and the 16S rDNA sequences of the determined strains were compared with the 16S rDNA/rRNA sequences of the known bacteria in the GenBank database for homology analysis, and the results of the analyses are shown in Table 5, Table 6, Table 7 and Table 8.

As can be seen from Table 5, Table 6, Table 7 and Table 8, strain 9# showed the highest homology with *Lactobacillus casei* with 99% similarity. Strain 16# showed the highest homology with *Lactobacillus tablets* with 95% similarity. Strain 23# showed the highest homology with *Lactobacillus plantarum* with 99% similarity. Strain 93# showed the highest homology with *Lactobacillus casei* with 99% similarity. Therefore, it can be tentatively determined that strains 9#, 23#, and 93# belong to the genus *Lactobacillus*, while 16# may belong to the genus *Schizococcus*.

#### 3.6.3. Construction of Phylogenetic Trees

The sequences of the sequenced strains were aligned with the 16Sr DNA sequences of the model strains selected from GenBank/EMBL/DDBJ/LPSN databases using MEGA4 software. Phylogenetic trees were constructed by the “Neighbor-Join method”. The reference strains of *Lactobacillus* used to construct the phylogenetic trees are shown in Table 9.

**Figure 5:**
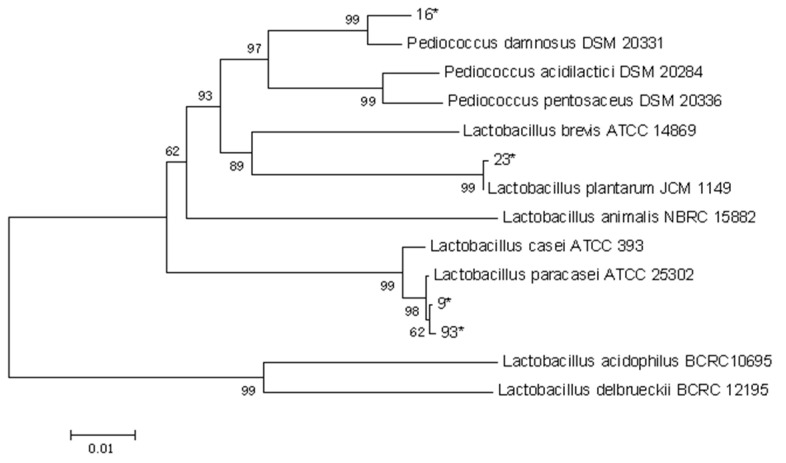
16s rDNA phylogenetic tree of strains. * means the numbering of the pairs of strains, 16*, 23*, 9* and 93* representing respectively 16#, 23#, 9# and 93#.

The phylogenetic tree drawn from the 16S rDNA sequences of strains 9#, 16#, 23#, and 93# with 10 lactobacilli of known taxonomic status is shown in Figure 5.

Figure 5 shows that these four strains can be categorized into two major groups. *Lactobacillus acidophilus* BCRC 10695 and *Lactobacillus delbrueckii* BCRC 12195 comprise the first cluster. The remaining four strains formed another group. It can be seen in the figure that strains 9# and 93# are genetically closest to *Lactobacillus paracasei* ATCC 25302, which can be categorized as *Lactobacillus paracasei*. Strain 23# was classified as *Lactobacillus plantarum*. The phylogenetic tree of strains 9#, 23#, and 93# showed results consistent with the comparative results of BLAST. When the sequence of 16# was submitted to GenBank for homology comparison, it showed the highest similarity with *Pediococcus acidilactici*, but this was much lower than the 99% similarity of other strains, and the results of constructing a phylogenetic tree with the sequences of the model strains showed that 16# had the closest genetic distance to *Pediococcus damnosus*, so it was not possible to determine which species belonged to the genus *Pediococcus*. When the phylogenetic tree was constructed together with the model strain, the result showed that strain 16# was the closest to *Pediococcus damnosus*, which did not match with the BLAST result, so it was not possible to determine which species belonged to the genus Pediococcus.

### 3.7. Determination of Strain Growth Curve

**Figure 6:**
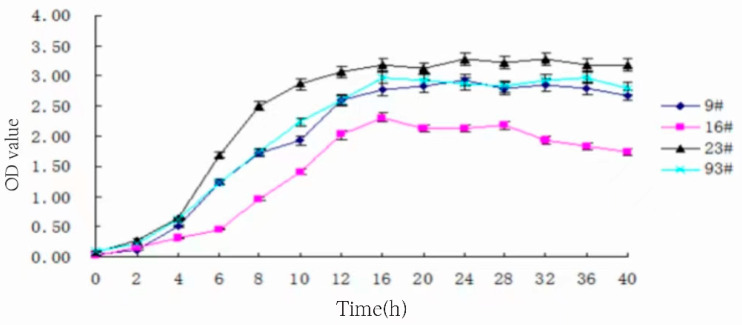
Strain growth curve.

The selected four strains were activated and inoculated in MRS liquid medium at 3% inoculum and incubated at 37 °C. Samples were taken at 0, 2, 4, 6, 8, 10, 12, 16, 20, 24, 28, 32, 36, and 40 h, respectively, and the OD value of the fermentation broth was measured at 600 nm, and the growth curves of the strains were plotted as shown in Figure 6.

The growth trend of the four strains is basically the same as can be seen in Figure 6. Moreover, 0 to 4 h was a delayed period; a possible reason for this was that the strains were adapting to the new growth environment during the pre-culture period, and the volume of bacteria did not rise substantially. The logarithmic period was from 4 to 16 h. The growth of lactic acid bacteria was fast, and the curve rose rapidly. Furthermore, 16 h later, it entered the stabilization period, and the OD value of the medium basically remained unchanged, and the growth and death of the bacteria were in a dynamic equilibrium. Among them, 9#, 23#, and 93# grew better, 9# and 93# grew basically the same, 16# was slightly worse in general, and the OD value fluctuated a lot in the late stage of fermentation, and there was a trend of gradual decline at the end.

### 3.8. Determination of Acidifying Capacity of Strains

**Figure 7:**
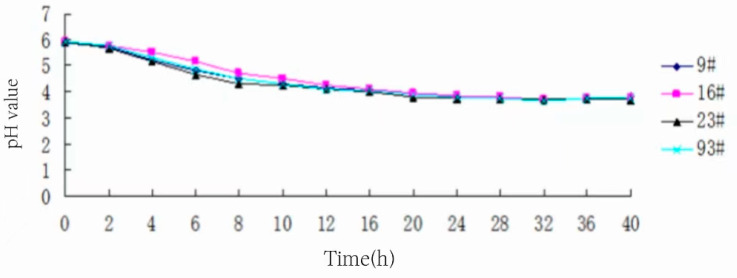
Trend of pH change in strains.

The four strains of bacteria were inoculated in MRS liquid medium at 3% inoculum and incubated at 37 °C. Samples were taken at 0, 2, 4, 6, 8, 10, 12, 16, 20, 24, 28, 32, 36, and 40 h, respectively, and the pH value of the medium was measured, and the curves of the pH value over time are shown in Figure 7.

Figure 7 shows that the trend of pH value change with incubation time of the four strains of bacteria is basically the same. It is slightly different only in the pre-growth period of acid production, and 16# acid production is weaker, 9# and 93# are basically the same, and 23# is slightly stronger. The pH value slowly decreased from 0 to 16 h and then gradually stabilized. Eventually, the pH stabilized at about 4. It can be concluded that the strain was in the logarithmic phase of vigorous growth before 16 h, with a corresponding high acid production capacity and a decreasing trend in pH. After 16 h, it entered the stabilization phase, and the pH value tended to be unchanged.

### 3.9. Determination of Optimum Temperature for Strain Growth

**Figure 8:**
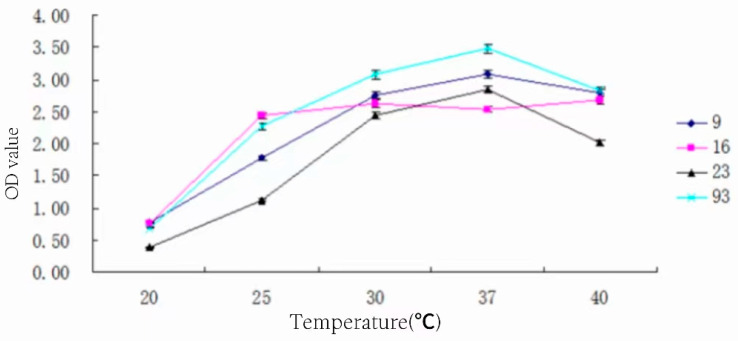
OD values of the medium of four strains of bacteria at different temperatures.

The strain was inoculated with 3% inoculum in MRS medium and placed in temperatures of 20 °C, 25 °C, 30 °C, 37 °C, and 40 °C for 24 h. The optimal growth temperature of the strain is shown in Figure 8.

From Figure 8, it can be seen that the optimum growth temperature of strains 9#, 23#, and 93# are all 37 °C, and the OD value of strain 16# remains basically unchanged in the medium from 25 °C to 40 °C. Strain 23# is more sensitive to temperature changes and does not tolerate high temperatures. Strain 16# has a gentle trend of change in the range of 25 °C~40 °C, indicating that the bacteria can grow stably in this temperature range.

### 3.10. Determination of Optimum pH for Strain Growth

**Figure 9:**
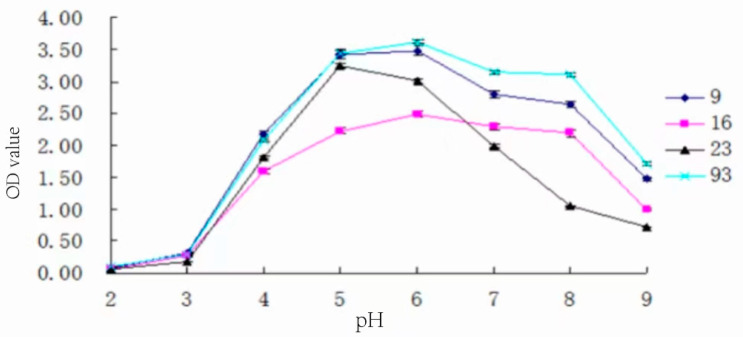
Growth of strains at different pH values.

The strains were inoculated with 3% inoculum in MRS medium at different pH values, respectively, and left to incubate at 37 °C for 24 h. The growth of the strains at different pH values is shown in Figure 9.

From Figure 9, it can be seen that the optimal pH of strains 9#, 93#, and 16# was 6, while the optimal pH of 23# was 5. The OD values of the medium of 9#, 16#, and 93# changed slowly within the range of pH 5–8, while the OD value of the medium of 23# varied drastically throughout the range of experimental pH, which indicated that the change in the medium pH had a greater impact on the growth of strain 23#. Although the overall growth condition of 16# was poorer than the other three strains, its optimal pH range was wider than the other three strains, and it could grow well at pH 4~8, suggesting it was more adaptable to pH changes. Strains 9# and 93# have both a higher growth ability and stronger adaptability to pH changes.

### 3.11. Salt Tolerance of Strains

**Figure 10:**
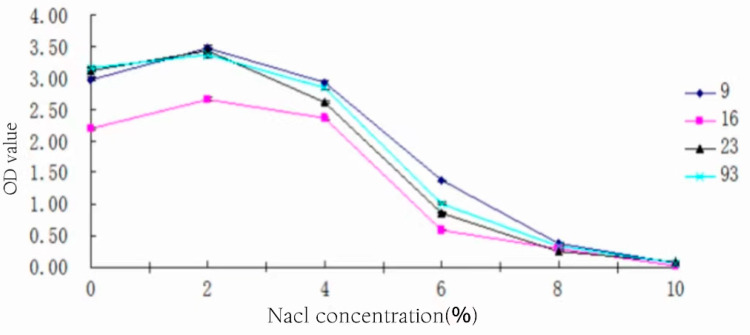
Salt tolerance of strains.

The strains were inoculated in MRS medium containing different proportions of NaCl at 3% inoculum, respectively, and left to incubate at 37 °C for 24 h. The salt tolerance of each strain is shown in Figure 10.

From Figure 10, it can be seen that the optimal salt concentration for the growth of all four strains is 2%, and the OD values of all four strains show a sharp decrease when the salt concentration is greater than 4%. The salt concentration of 10% basically shows no growth. However, it shows when it is less than 2%, the increase in salt concentration also plays a certain role in promoting the growth of bacteria. Strain 9# grew slightly better than the other three strains at high salt concentrations.

### 3.12. Determination of Acid Resistance

**Figure 11:**
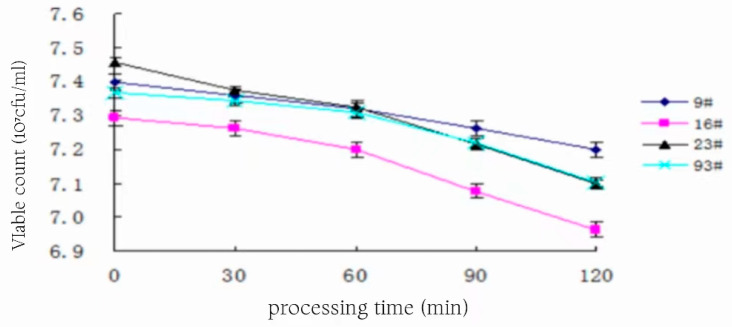
Number of viable bacteria of the strain at pH 3.0.

**Figure 12:**
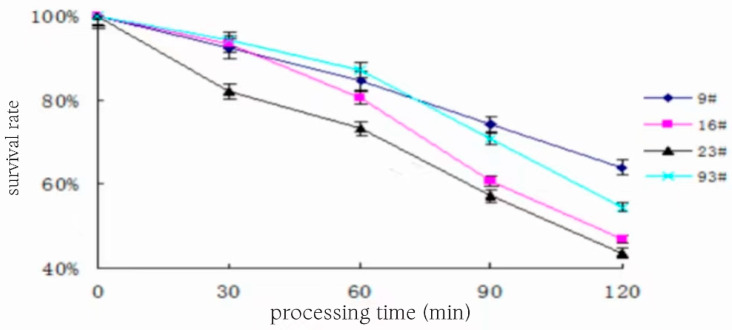
Survival of strains at pH 3.0.

The pH 7.4 PBS buffer with hydrochloric acid was used to adjust the pH to 3.0, and the activated bacterial solution was obtained at an inoculum of 10% after sterilization at 121 °C and 15 min, and the changes in the number of viable bacteria of the four strains are shown in Figure 11, and the survival rate is shown in Figure 12, after acting for different times at 37 °C, respectively.

As can be seen in Figure 11 and Figure 12, the logarithmic value of the number of viable bacteria as well as the survival rate of strain 9# at pH 3.0 showed a slow decreasing trend with the prolongation of the treatment time, and after 120 min of treatment, the number of viable bacteria was 1.58 × 107, and the logarithmic value of the number of viable bacteria was 7.199, with a survival rate of 64%. The logarithmic value of the number of viable bacteria and the survival rate of strain 23# changed with the prolongation of the treatment time, showing that a rapid decline in the trend of the number of viable bacteria was 1.25 × 10^7^, the logarithmic value of the number of viable bacteria was 7.097, and the survival rate was only 44% after the treatment for 120 min under the condition of pH 3.0. The trend of the change in 93# and 16# was in the middle of the above two. Overall, bacterial number 9# had better acid tolerance than the other three strains and could survive well under low pH conditions similar to human gastric juice to fulfill the function of probiotics. Among the four strains, 9# had stronger growth ability as well as acid and salt tolerance, so 9# was chosen as the experimental strain in the subsequent experiments of optimizing the conditions for CLA production by fermentation.

### 3.13. Effect of Medium Type on CLA Production

**Figure 13:**
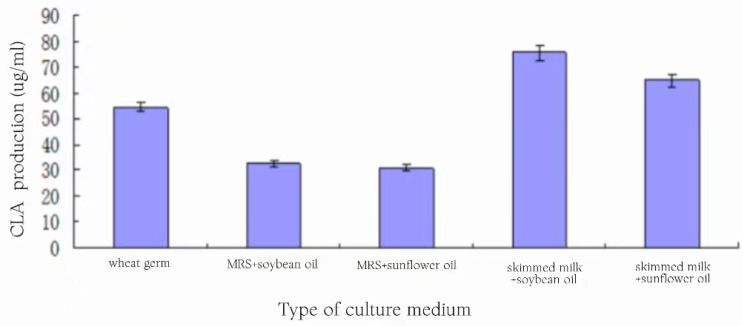
Levels of CLA production by fermentation in different media.

A total of five media of two types were selected for the experiment, each with an inoculum of 3%, and left to incubate at 37 °C for 24 h. The yield of CLA produced by the fermentation of each media is shown in Figure 13.

Upon analyzing Figure 13, it could be seen that strain 9# could produce CLA by fermentation in all five media, and the CLA yield of this group of media with MRS-added substrate was on the low side, which was around 30 μg/mL. And, the CLA yield of the wheat germ medium without the substrate addition was higher than that of the MRS group but lower than that of the skimmed milk plus substrate group. The highest yield of 75.51 ± 2.77 μg/mL was achieved by skimmed milk medium supplemented with soybean oil emulsion, and the yield of skimmed milk medium supplemented with sunflower oil was slightly lower at 64.74 ± 2.45 μg/mL.

It appears that the skimmed milk medium with the addition of vegetable oil emulsion has a higher CLA production capacity, probably because this medium combines the advantages of the other two groups of media. On the one hand, it is richer in nutrients than the MRS medium, especially proteins, amino acids, trace elements and other substances, which may promote lactic acid bacteria growth and product metabolism [24,25]. On the other hand, the linoleic acid in wheat germ is not completely soluble in the medium, while the vegetable oil emulsion added to skim milk can be completely soluble in the medium, which makes the substrate content of the skim milk medium higher than that of the wheat germ medium and promotes the activity of the linoleic acid isomerase enzyme in the body of the lactobacilli and the conversion of generating a larger amount of CLA. Also, Figure 13 shows that the amount of CLA produced by the fermentation of the medium with soybean oil as a substrate was slightly higher than that of sunflower oil; the reason may be due to the higher LA content in soybean oil than sunflower oil. Therefore, the skimmed milk medium with soybean oil emulsion as the substrate was chosen as the fermentation medium for the subsequent experiments.

### 3.14. Effect of Substrate Addition on CLA Yield

**Figure 14:**
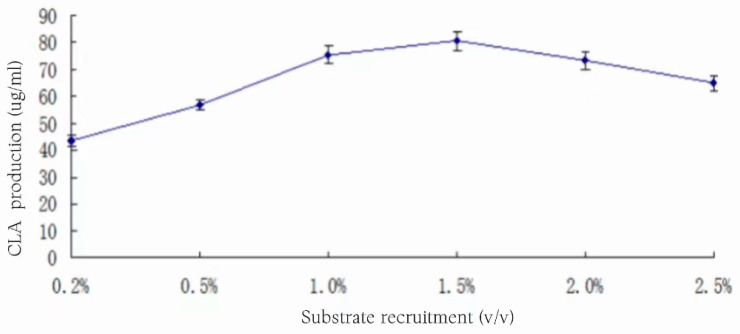
Effect of substrate addition on CLA yield.

The substrate soybean oil emulsion with volume fractions of 0.2%, 0.5%, 1%, 1.5%, 2%, and 2.5% was added to the medium with an inoculum volume of 3%, and the incubation was left to incubate at 37 °C for 24 h. The effect of substrate additions on the yield of CLA is shown in Figure 14.

Upon analyzing Figure 14, it can be seen that the CLA yield increases and then decreases with the increase in substrate addition. The CLA yield increased rapidly when the substrate addition was less than 1.0%, increased slowly between 1.0% and 1.5%, and reached a maximum value of 80.54 ± 3.87 μg/mL when the substrate addition was 1.5%, and then it decreased slowly when the substrate addition was increased. The reason for the decrease in yield may be that the presence of substrates has a certain inhibitory effect on the ability of the strain to convert to produce CLA, but the strain has a certain tolerance to the inhibitory effect of substrates, and even at a substrate concentration of 2.5%, the yield of CLA can still reach 64.93 ± 2.79 mg/mL, which is 81% of the highest yield. Therefore, the substrate addition of 1.5% was selected for fermentation in the subsequent experiments.

### 3.15. Effect of Inoculum Size on CLA Yield

**Figure 15:**
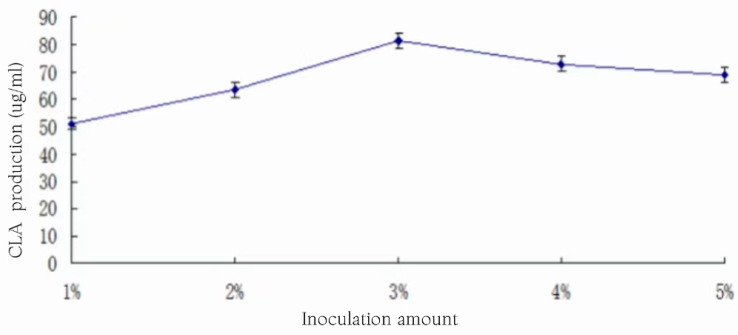
Effect of inoculum size on CLA production.

The medium was inoculated with inoculation amounts of 1%, 2%, 3%, 4%, and 5%, and the substrate concentration was 1.5%, and the incubation took place at 37 °C for 24 h. The effect of the inoculum amount on the yield of CLA is shown in Figure 15.

Figure 15 shows that with the increase in inoculum amount, CLA yield showed a trend of increasing and then decreasing. The nutrients were enough for lactic acid bacteria to be utilized in the case of the inoculum amount less than 3%, but the overall number of bacteria was low, so the CLA yield was not high. When the inoculum amount was 3%, the strain had the largest CLA yield, reaching 81.33 ± 3.01 μg/mL. The CLA yield began to decrease when the inoculum amount was increased, which could be attributed to the fact that when the inoculum amount was increased, there were many nutrients in the medium, and the nutrients in the medium were rapidly consumed, which affected the CLA yield [26]. Therefore, the subsequent experiments selected an inoculum level of 3% for fermentation.

### 3.16. Effect of Fermentation Time on CLA Yield

**Figure 16:**
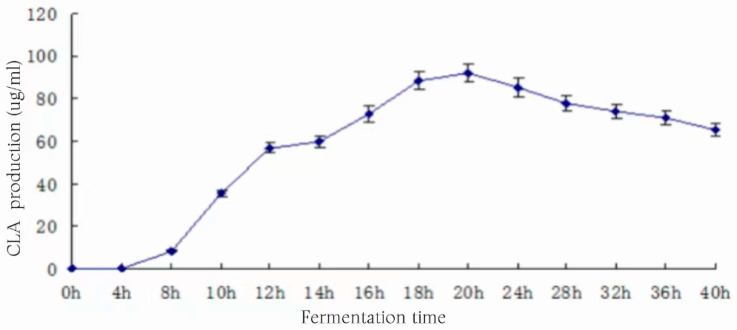
Effect of fermentation time on CLA yield.

The medium was inoculated at 3% and the substrate concentration was 1.5% (*v*/*v*) and incubated at 37 °C for 40 h. Samples were taken at regular intervals to check the CLA content, and the relationship between the fermentation time and the CLA production is shown in Figure 16.

As analyzed in Figure 16, there was no CLA production in the fermentation broth from 0 to 4 h after inoculation, and CLA production began to increase rapidly from 8 h onwards, reaching a maximum yield of 91.84 ± 4.33 μg/mL at 20 h. After that, the amount of CLA production gradually decreased with the extension of fermentation time. The growth curves of the previous strains showed that 0 to 4 h was the growth retardation period of the bacteria. At this time, the microorganisms utilized a large number of nutrients in the medium for the value-added bacteria, and metabolites had not yet begun to accumulate, which may have been the reason for the absence of CLA generation in the first 4 h of fermentation. Fermentation from 4 to 16 h was the logarithmic period of bacterial growth; bacterial growth and product metabolism were in the peak period, and the CLA content in the fermentation broth continued to increase. After 20 h, the bacterial growth entered the stabilization period, and the metabolism entered a relatively stable period because the biosynthesis of CLA was a dynamic process. Additionally, CLA is not a metabolism end-product but an intermediate product of the biohydrogenation process, which cannot be accumulated in large quantities and continues to be hydrogenated to generate oleic acid or stearic acid, so there will be a slow decrease in CLA content [27,28]. Therefore, 20 h was chosen as the appropriate fermentation time for the subsequent experiments.

**Figure 17:**
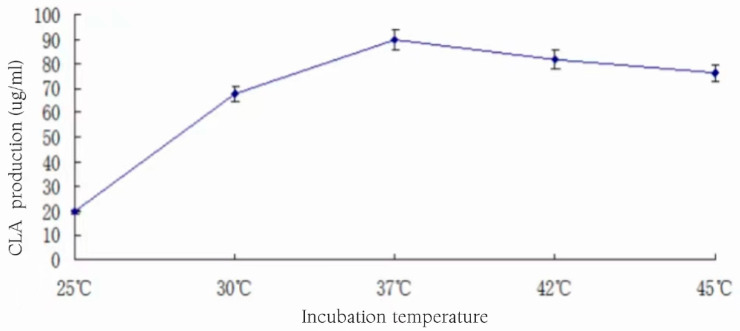
Effect of incubation temperature on CLA production.

From the analysis of Figure 17, it could be seen that with the rise in culture temperature, CLA production showed a trend of increasing and then decreasing, and it was concluded that 37 °C was the optimal growth temperature for the bacterium, and it also showed that CLA production was the highest at 37 °C, and the bacterium metabolized vigorously under the optimal growth temperature, and the conversion rate of CLA was higher, reaching 89.77 ± 4.32 μg/mL. Lactobacilli did not produce high CLA at 25 °C and 30 °C due to non-optimal growth conditions, and the reason for the slow decrease in production at temperatures higher than 37 °C may be due to the weakening of lactobacilli growth by temperature on the one hand, along with the decrease in the accumulation of metabolites, and on the other hand, higher temperatures also favored the conversion of CLA to other substances, which resulted in a decrease in the content of CLA in the fermentation broth [29]. Therefore, the subsequent experiments were chosen to carry out the fermentation at 37 °C.

### 3.17. Effect of Initial pH on CLA Production

**Figure 18:**
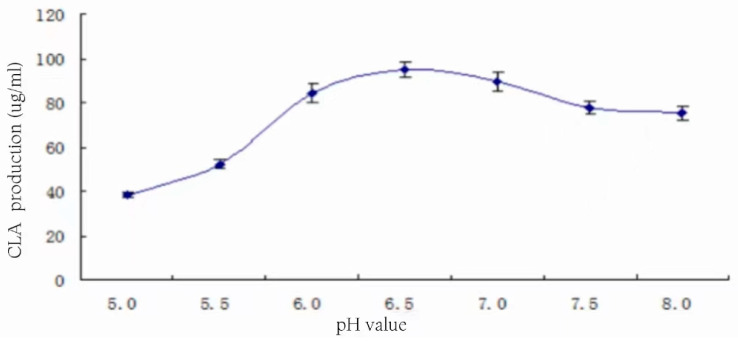
Effect of initial pH on CLA production.

The effect of initial pH on CLA production is shown in Figure 18, which is based on 3% inoculum obtained in different pH media with a substrate concentration of 1.5% (*v*/*v*) and 20 h of stationary incubation at 37 °C.

From the analysis of Figure 18, CLA yield showed a trend of increasing and then decreasing with the rise in the initial pH. In the range of pH 6–7, the CLA production varied very little, and the highest production was 94.68 ± 3.57 μg/mL at pH 6.5, which was also very close to the pH of skimmed milk itself, indicating that the natural pH of the medium was very suitable for the transformation of the strain to produce CLA.

## 4. Conclusions and Recommendations

In this experiment, four strains with high CLA-producing ability were screened from different brands of yogurt and kimchi through a series of isolation and screening. Among them, strain 9# and strain 16# were screened from Xiaojuan sauerkraut, and strain 23# and strain 93# were screened from Dongzhi yogurt. The four strains screened were identified using a combination of traditional morphological and physiological biochemical analyses and 16Sr DNA sequence analysis, which resulted in the classification of strains 9# and 93# as *Lactobacillus paracasei*, strain 23# as *Lactobacillus plantarum*, and strain 16# as *Schizosaccharomyces cerevisiae* spp. Among them, strain 9# had a relatively strong ability to produce conjugated linoleic acid of 54.28 ± 2.12 μg/mL, and by optimizing the experimental conditions, the final yield of conjugated linoleic acid converted by strain 9# could reach 94.68 ± 3.57 μg/mL.

## Figures and Tables

**Table 1:** The absorbance value of the initial screening strain at 233 nm.

Strain Number	Absorption Value	Strain Number	Absorption Value	Strain Number	Absorption Value
1#	0.182	35#	0.406	68#	0.229
8#	0.288	38#	0.504	70#	0.325
9#	0.807	43#	0.401	73#	0.311
10#	0.546	47#	0.271	75#	0.25
12#	0.492	48#	0.58	80#	0.481
16#	0.797	55#	0.156	81#	0.158
19#	0.115	56#	0.321	89#	0.624
23#	0.821	60#	0.249	92#	0.405
26#	0.392	64#	0.529	93#	0.805
30#	0.381	66#	0.231	95#	0.307
34#	0.121	67#	0.428		

This section.

**Table 2:** Fermentation CLA yield of each strain.

Strain Number	CLA Content (µg/mL)	Strain Number	CLA Content (µg/mL)
8#	4.72	64#	24.96
9#	54.28	66#	1.44
10#	11.12	67#	14.89
16#	49.29	68#	2.10
12#	24.02	70#	1.68
23#	45.83	73#	15.84
38#	29.47	75#	1.47
43#	21.50	89#	26.64
55#	1.26	92#	16.15
56#	6.72	93#	34.82

**Table 3:** Physiological and biochemical test results of strains.

Identification Project	9#	16#	23#	93#
Catalase	−	−	−	−
Gelatin liquefaction	−	−	−	−
Nitrate reduction	−	−	−	−
Hydrogen sulfide production	−	−	−	−
Litmus milk produces acid	+	+	+	+
Stone stamen milk decolorization	+	+	+	+
Litmus milk curd	+	+	+	+
Liquefaction of litmus milk	−	−	−	−

Note: “+” strain positive; “−” strain negative.

**Table 4:** Sugar fermentation test results of strains.

Fermented Sugars	9#	16#	23#	93#	Fermented Sugars	9#	16#	23#	93#
glucose	+	+	+	+	sucrose	+	+	+	+
fructose	+	+	+	+	inulin	−	−	−	−
lactose	+	+	+	+	melibiose	−	−	+	−
galactose	+	−	+	+	rhamnose	−	−	−	−
maltose	+	+	+	+	sorbitol	+	−	+	+
xylose	−	−	−	−	sorbic acid	−	−	−	−
mannose	−	−	−	−	arabinose	−	−	+	−
mannitol	+	−	+	+					

Note: “+” strain positive; “−” strain negative.

**Table 5:** Comparison of the 16S rDNA homology of strain 9#.

Registry Number	Strain Name	Score	Similarity
NC_014334	*Lactobacillus casei*	2639	99%
NC_010999	*Lactobacillus casei*	2639	99%
NC_008526	*Lactobacillus casei*	2639	99%

**Table 6:** Comparison of the 16S rDNA homology of strain 16#.

Registry Number	Strain Name	Score	Similarity
AGKB01000006	*Pediococcus acidilactici*	2296	95%
AEEG01000012	*Pediococcus acidilactici*	2290	95%
ACXB01000026	*Pediococcus acidilactici*	2285	95%

**Table 7:** Comparison of the 16S rDNA homology of strain 23#.

Registry Number	Strain Name	Score	Similarity
NC_014554	*Lactobacillus plantarum*	2638	99%
NC_012984	*Lactobacillus plantarum*	2638	99%
ACGZ02000033	*Lactobacillus plantarum*	2632	99%

**Table 8:** Comparison of the 16S rDNA homology of strain 93#.

Registry Number	Strain Name	Score	Similarity
NC_014334	*Lactobacillus casei*	2612	99%
NC_010999	*Lactobacillus casei*	2612	99%
NC_008526	*Lactobacillus casei*	2612	99%

**Table 9:** Reference strains of lactic acid bacteria for constructing phylogenetic trees.

GenBank Registration Number	Strain Name	Strain Number
AF469172	*Lactobacillus casei*	ATCC 393
AY773949	*Lactobacillus delbrueckii*	BCRC 12195
HQ423165	*Lactobacillus paracasei*	ATCC 25302
HM162417	*Lactobacillus plantarum*	JCM_1149
AY773947	*Lactobacillus acidophilus*	BCRC 10695
EU194349	*Lactobacillus brevis*	ATCC 14869
AJ305320	*Pediococcus acidilactici*	DSM 20284
AJ305321	*Pediococcus pentosaceus*	DSM 20336
AJ318414	*Pediococcus damnosus*	DSM 20331

## Data Availability

The original contributions presented in the study are included in the article, further inquiries can be directed to the corresponding author.
